# Thrombopoietic stimulating activity of rhTyrRS (Y341A)

**DOI:** 10.1038/s41598-017-12073-4

**Published:** 2017-09-22

**Authors:** Yu Jinchao, Zhang Yanling, Wang Xu, Zhao Bing, Ye Yuhao, Zhou Weiran, Sun Shaoyang, Ma Liyun, Shi Yun, Zhan Ling, Yu Min, Mo Wei

**Affiliations:** 1 The Key Laboratory of Metabolism and Molecular Medicine, Ministry of Education, Fudan University, 238# P.O. Box, 138 Yixueyan Rd., Shanghai, 200032 Shanghai Shi China; 2 The Department of Biochemistry and Molecular Biology, School of Basic Medical Sciences, Fudan University, 238# P.O. Box, 138 Yixueyan Rd., Shanghai, 200032 Shanghai Shi China; 3 Collaborative Innovation Center for Biotherapy, Sichuan University, Chengdu, China

## Abstract

Tumor radiotherapy induces hematopoietic organ damage and reduces thrombocyte counts. Thrombocytopenia is a common disease. Some studies have shown that tRNA synthetase plays not only catalytic tRNA aminoacylation roles, but also functions similarly to cytokines. Recombinant human tyrosyl-tRNA synthetase with a mutated Y341A (rhTyrRS (Y341A)) promotes megakaryocyte migrate from bone marrow to peripheral blood. It would promote megakaryocytes in the lungs adhering to vascular endothelial cells and resulting in the platelet production. The purpose of this research was to investigate the efficacy of rhTyrRS (Y341A) as a therapy for thrombocytopenia and to explore its mechanism of action. We found platelet number was effectively increased by rhTyrRS (Y341A) via platelet count and reticulated platelets (RPs) flow cytometry. We also demonstrated radiation-induced thrombocytopenia could be prevented by rhTyrRS (Y341A). The results of immunohistochemistry and H&E staining showed the number of pulmonary mature megakaryocytes was significantly increased in rhTyrRS (Y341A) treated groups. In transgenic zebrafish larvae, confocal microscopy results showed rhTyrRS (Y341A) promoted the migration and adhesion of megakaryocytes. These results suggested that rhTyrRS (Y341A) promote megakaryocytes in bone marrow migrating to lungs through blood circulation. rhTyrRS (Y341A) may be an effective medicine which could be used to treat patients suffering from thrombocytopenia.

## Introduction

Thrombocytopenia is a common clinical disease, frequently induced by radiation therapy or chronic liver disease. Radiotherapy is especially harmful and can cause significant blood cell damage or reduced counts, which includes significant detrimental effects on platelets.

Bone marrow has been proposed to be a major site of platelet production, however, recently Emma lefrançais’s experiments proved that the lung was a site of platelet biogenesis^1^. Our previous study showed that rhTyrRS (Y341A) promoted megakaryocyte migration from bone marrow to peripheral blood^2,3^. These results suggest that rhTyrRS (Y341A) may also promote the migration of megakaryocytes to the lungs through the circulation of the blood. Meanwhile, we speculate that rhTyrRS (Y341A) promotes megakaryocytes in the lungs adhering to vascular endothelial cells, resulting in the platelet production.

Aminoacyl-tRNA synthetases (AARSs) are a group of proteins that are highly conserved across species. They are found in animals, plants, bacteria, viruses, and other organisms. AARSs assist amino acid transfer to the corresponding tRNA and participate in protein synthesis, which contributes to genetic diversity in these organisms. Because these reactions require the capacity to recognize tRNA, as well as small chemicals, such as amino acids and ATP, the structures of AARSs are well equipped for interacting with diverse molecules, which is likely associated with their functional versatility^4^.

Amino acid structures are largely conserved across species. However, tRNA and their corresponding AARSs have changed greatly over the course of evolution and vary among organisms. The structures of AARSs and tRNA show high species specificity, associated with their specific biological functions. Therefore, the structure and function of AARSs has become a new/important focus of study^5^.

The latest research shows that AARSs not only take part in protein synthesis, but they also participate in various life activities, including transcription and translation regulation, RNA splicing, signal transduction, and the immune response. Wakasugi and Schimmel discovered that the human tyrosyl-tRNA synthase (TyrRS) was hydrolyzed into two fragments by elastase: a C-terminal fragment and N-terminal fragment (Mini TyrRS)^6^.

The C-terminal fragment is similar to endothelial activation peptide EMAP II, as far as its structure and function. It not only induces the mononuclear phagocytic system (MPs) and polymorphonuclear leukocyte (PMN) migration^7^, but it also stimulates MPs to produce tumor necrosis factor and stimulates PMNs to release myeloperoxidase. However, the full-length TyrRS does not possess cytokine functions^5,8,9^, only the C-terminal domain functions as a cytokine.

The N-terminal fragment has aminoacylation abilities, functioning much like the IL-8 cytokine^2^. It promotes angiogenesis, which mainly relies on a three amino acid motif composed of Glu-Leu-Arg (ELR). An ELR mutation will lead to mini TyrRS cytokine loss of function, while the full-length TyrRS loses its IL-8 chemokine function^10,11^. Based on the crystalline structure of mini TyrRS, the ELR motif is in the catalytic domain of the alpha helix 5^12^. Y341 in the alpha 14 spiral in the hydrogen bond network plays a key role; destruction of the hydrogen bond network causes the alpha helix 5 to be released. Thus, the ELR motif is exposed and plays roles similar to cytokines^13–15^. The N-terminal fragment of the TyrRS also acts as a chemoattractant molecule. TyrRS facilitates the maturation of megakaryocytes, which causes an increase in platelet production^16–18^. It has been predicted that the C-terminus of the full-length protein is tethered to the N-terminal region, thus preventing ELR substrate recognition^9,19^. A full-length human tyrosyl-tRNA synthetase with a mutated Y341 (tyrosine mutated to alanine) causes the ELR motif to be exposed, such that the mini-rhTyrRS promotes thrombocytopoiesis. A single tyrosine was changed to an alanine at position 341 to unmask the ELR motif, which delivers its thrombopoietic activity^18,19^. As a potential thrombocytopoietic drug, we studied rhTyrRS (Y341A) in the hope of using it as an adjuvant to radiotherapy; we further examined cell migration and adhesion and demonstrated that rhTyrRS (Y341A) acts as a chemoattractant for M-07e cells^7,18–20^.

Due to its small size, optical transparency, high fecundity, and similarity to humans (as far as genetics and anatomy are concerned), zebrafish (*Danio rerio*) serve as a novel mainstream vertebrate model with significant advantages for studying developmental genetics and human diseases^20,21^. Recently, zebrafish have also become a valuable tool for investigating the novel functions of small molecule compounds and have been used as a platform for high-throughput targeted drug screening^22^. Zebrafish also represent an ideal vertebrate model for cardiovascular system research^23,24^. Furthermore, mammalian megakaryocytes are homologs of non-mammalian thrombocytes that evolved during the course of vertebrate evolution^25^. Therefore, we used zebrafish to examine the effects of rhTyrRS (Y341A).

The CD_41_ antigen is a glycoprotein with a relative molecular mass of 14,000 Da. CD_41_ is an integrin αIIb chain, also known as platelet glucoprotein II b (GP II b). It is located on the surface of megakaryocyte/platelet progenitors, as well as on mature platelets. A transgenic reporter zebrafish using the CD_41_ promoter to drive enhanced green fluorescent protein (EGFP) expression was previously generated, through which green fluorescent megacaryocytes could be observed in the zebrafish at 48 hours post fertilization (hpf)^26,27^.

Zebrafish express vascular endothelial growth factor (VEGF) and its receptor Flt-1^27,28^. The biological activity of VEGF is conferred mainly through two structures related to the high affinity tyrosine kinase receptor: VEGFR-1 (FMS like tyrosine kinase, Flt-1) and VEGFR-2 (kinase domain receptor/fetal liver kinase receptor 1, KDR/Flk-1)^29^. Although VEGFR-1 has high affinity for VEGF^30^, VEGFR-2 is the main mediator of VEGF functions in the cell. VEGFR-2 (Flk-1) gene deficient mice will die during embryonic development (day 8.5 to 9.5) due to defects in hematopoietic and vascular endothelial cells^31,32^. Therefore, *in vivo* VEGF mainly signals through VEGFR-2. Zebrafish vascular distribution and development can be clearly observed when VEGFR-2 (Flk-1) is marked by a fluorescent protein. A transgenic reporter zebrafish using *flk1* promoter to drive red fluorescent protein (RFP) expression was previously generated, in which the vascular endothelial could then be observed in the zebrafish at 48 hpf^33^. With the help of these two types of transgenic zebrafish, we can easily carry out the research on rhTyrRS (Y341A) *in vivo*.

## Materials and Methods

### Reagents

The human umbilical vein endothelial cell (HUVEC) line was purchased from the Institute of Biochemistry, Chinese Academy of Sciences cell library. RhTyrRS (Y341A) and zebrafish cDNA library were provided by our laboratory. Anti-ITGA2B (integrin, alpha 2b) was purchased from Boster Bio. The Nuclear transcription factor κB (NF-κB (p65)) monoclonal antibody and the Alexa 488-conjugated IgG antibody were purchased from Molecular Probes.

Thrombopoietin (TPO) was purchased from 3SBIO INC China. PTU (1-phenyl-2-thiourea) and Tticaine (3-Aminobenzoic Acid Ethyl Ester Methanesulfonat) Thiazole orange (TO), Phalloidin and DAPI were purchased from Sigma-Aldrich. Texas Red-X was purchased from GeneSeq Tools, while the mMESSAGE mMACHINE^®^ T7 was purchased from ThermoFisher, and the RNAclean Kit were purchased from TIANGEN.

### Preparation of zebrafish TPO mRNA

We designed primers to extract the zebrafish TPO gene from zebrafish cDNA library. Zebrafish TPO mRNA was obtained using the mMESSAGE mMACHINE T7 kit *in vitro*, and TPO mRNA was purified using an RNAclean Kit (250 ng/μl).

### Animals

Zebrafish embryos, larvae, and adult fish were raised under standard laboratory conditions at 28.5 °C. The spawn of zebrafish with CD_41_, marked by green fluorescence protein (GFP) in the megakaryocyte *Tg* (*CD*_41_ *: EGFP*)^34^, and Flk-1 marked by red fluorescence protein (RFP) in the vascular endothelial cell *Tg* (*flk1: mCherry*)^33^, were provided in our laboratory. The *Tg (CD*_41_ *: EGFP)* line was a generous gift from Professor Weijun Pan at the Institute of Health Sciences, Shanghai Institutes for Biological Sciences. At 20 hpf, 4 ml 1-phenyl 2-thiourea (PTU) was added to prevent the formation of melanin in the zebrafish.

Sprague Dawley (SD) rats (300 ± 30 g, 50% male and 50% female), and C57BL/6 mice (8–10 weeks, 50% male and 50% female) were purchased from the Animal Center of Fudan University. All animals were kept in cages at 24 °C and 65% humidity, with an alternating 12:12 h light/dark cycle and allowed free access to food and tap water in accordance with the Guide for the Care and Use of Laboratory Animals (Fudan University). All studies involving animal manipulations were approved by the Fudan University Shanghai Medical College Animal Care and Use Committee and followed the National Institutes of Health guidelines for the care and use of animals.

### Platelet counts

30 SD rats (300 g, 50% male and 50% female) or 30 C57BL/6 mice (25 g, 50% male and 50% female) were injected intraperitoneally with rhTyrRS (Y341A) (1 μg/kg), rhTyrRS (Y341A) (10 μg/kg), rhTyrRS (Y341A) (100 μg/kg), TPO (300U/kg), or PBS for 3 consecutive days (n = 6 per group). Blood samples were collected from the caudal vena cava, and sodium citrate (3.8%) was used as the anti-coagulant (1:9). The number of blood cells was measured by a Mindray automatic blood cell analyzer (BC-2800). Platelet counts were measured for other 7 days followed with 3-days administration.

### Flow Cytometry

30 SD rats (300 g, 50% male and 50% female) or 30 C57BL/6 mice (25 g, 50% male and 50% female) were injected intraperitoneally with rhTyrRS (Y341A) (100 μg/kg), TPO (300U/kg), or PBS for 3 consecutive days (n = 6 per group). On the day after the last treatment dose (study day 4), rat whole blood samples were collected through heart puncture and abdominal aortic blood sampling. Sodium citrate (3.8%) was used as the anti-coagulant (1:9). Approximately 5 μl of whole blood was added to a tube containing 1 ml of 10% thiazole orange (TO; Sigma). Flow cytometry was performed for 1 h to count at least 10,000 stained platelets within the platelet gate. The platelets demonstrating an increase in the mean fluorescence intensity beyond a threshold margin of 1% of the total platelets at baseline were counted as reticulated platelets (RPs). RPs were expressed as the percentage of the total counted platelets^35^.

### Radiation experiment in mice

Sixty C57BL/6 mice (25 ± 2 g) were randomly divided into pre-treatment and post-treatment groups. Mice in the pre-treatment and post-treatment groups were randomly divided into 5 groups (n = 6 per group) as follows: PBS group (negative control), rhTyrRS (Y341A) (1 μg/kg) group, rhTyrRS (Y341A) (10 μg/kg) group, rhTyrRS (Y341A) (100 μg/kg) group and TPO (300U/kg, positive control) group. Mice underwent irradiation (Cs137, 1 Gy/min) for two minutes. In the pre-treatment groups, mice were pre-treated rhTyrRS (Y341A) and TPO for three consecutive days before radiotherapy. In the post-treatment groups, after irradiation for three days, the mice were treated with rhTyrRS (Y341A) and TPO for another three consecutive days. The radiation experiment process is outlined in Table 1.

**Table 1:** Radiation experiment process.

Pre-treatment group				post-treatment group				
	D3-D1 before radiotherapy	D0	D1-D3 received radiotherapy		D1 before radiotherapy	D0	D1 received radiotherapy	D3-D5 received radiotherapy
PBS (negative control)	Mice were treated with PBS or rhTyrRS (Y341A) or TPO for three consecutive days before radiotherapy.	receive radiotherapy	Blood sampling in every day	PBS (negative control)	Blood sampling	receive radiotherapy	Blood sampling	Mice were treated with PBS or rhTyrRS (Y341A) or TPO for three consecutive days after radiotherapy.
rhTyrRS (Y341A) (1 μg/kg)				rhTyrRS (Y341A) (1 μg/kg)				
rhTyrRS (Y341A) (10 μg/kg)				rhTyrRS (Y341A) (10 μg/kg)				
rhTyrRS (Y341A) (100 μg/kg)	Blood sampling in every day			rhTyrRS (Y341A) (100 μg/kg)				Blood sampling in every day
TPO (300U/kg, positive control)				TPO (300U/kg, positive control)				

PBS, rhTyrRS (Y341A), or TPO were administered through intraperitoneal injection. Daily blood sampling was conducted for 1 week via tail bleeding. Mice were held in position in a 50 ml centrifuge tube, and then we cut the tail vein and used a micropipette to draw 30 µL of blood into an anticoagulant tube (3.8%Sodium citrate, 1:9). A Mindray automatic blood cell analyzer (BC-2800) was used to measure the number of platelets.

### Immunohistochemistry and histological analysis

Eighteen SD rats (300 g, 50% male and 50% female) were divided into 3 groups (n = 6 per group), and each group was injected intraperitoneally with PBS, rhTyrRS (Y341A) (100 μg/kg) or TPO (300 U/Kg) for three consecutive days. On the day after the last treatment dose (study day 4), the femurs and lungs were dissected out. The femurs decalcified using EDTA, then both the femurs and lungs were fixed in a 4% formaldehyde solution. Hematoxylin/eosin staining was performed on tissue using a standard protocol and consecutive sections to observe the number of megakaryocytes in the bone marrow and lung.

The following method was performed for immunohistochemistry. Paraffin-embedded longitudinal sections of the femurs and lungs were cut into 10 µm thick sections. After being posted on the slide, tissues were treated with xylene and deparaffinized. The sections were dehydrated in graded alcohol. After sections were rinsed in phosphate-buffered saline (PBS, pH 7.4), the sections were incubated with 3% hydrogen peroxide for 30 min. Sections were incubated with 5% goat serum for 30 min at room temperature (RT) to block non-specific binding sites, then sections were incubated with an anti-CD_41_ antibody (Abcam). Incubations with the primary antibody were conducted overnight at 4 °C, and then sections were washed three times with PBS and incubated with an HRP polymer conjugate (Invitrogen Corp.) for 1 hour at RT. Sections were subsequently washed three times with PBS and incubated with streptavidin peroxidase for 10 minutes. The same sections were washed and reacted with 3,3′-diaminobenzidine (DAB; DAKO Corp.) for 10 minutes at room temperature and counter-stained with Mayer’s hematoxylin. Sections were observed under a light microscope (Nikon, Tokyo, Japan).

In each 10 x microscope field, the megakaryocytes were quantified in the marrow of treated rats. Megakaryocyte counts were performed for 6 different 10 x microscope fields in each group, and the number of megakaryocytes was the denominator for the statistics expressed as mean ± SEM.

### Zebrafish assay

The selected male *Tg* (*CD41: EGFP*) zebrafish we crossed with female *Tg* (*flk1: mcherry*) zebrafish, and the embryos were bred into larvae. Two days post fertilization (dpf) larvae were selected and incubated in E3 solution in petri dishes. The filial generation Zebrafish, which was both labeled with red and green fluorescence under the dissecting microscope were chosen. Each green point in the peripheral blood represented a thromboblast that was labeled with green fluorescence, while the blood vessels were labeled with red fluorescence.

Sixty zebrafish were randomly divided into 5 groups (n = 12 per group): PBS, rhTyrRS (Y341A) (2 × 10^−1^ ng per fish, 2 × 10^−2^ ng per fish, 2 × 10^−3^ ng per fish), and TPO mRNA (0.05ng per fish) were microinjected into the zebrafish in 48 hpf; zebrafish were anesthetized with tricaine under a dissecting microscope (OLYMPUS DF PL APO 1X PF) and observed at 24 and 48 h. The adhesion conditions of thromboblasts to vascular endothelial cells in the zebrafish caudal hematopoietic tissue (CHT) after treatment with PBS, rhTyrRS (Y341A) (0.02 ng per fish), and TPO mRNA (0.05ng per fish) was further investigated via confocal microscopy (Leica LAS AF Lite).

### Cell immunofluorescence assay

HUVECs were seeded into 6-well plates (24 × 24 mm) in basal medium, and the medium was replaced with serum-free medium when the cells reached 70–80% confluency. Cells were stimulated with rhTyrRS (Y341A) (10 nM) or PBS for 2 hours at 37 °C. HUVEC cells were fixed with 4% paraformaldehyde for 10 minutes, then rendered permeable with 0.2% Triton X-100 for 10 min and blocked with 5% BSA for 30 min at RT. Cells were then incubated at 4 °C overnight with a NF-κB (p65) monoclonal antibody. The cells were washed three times with Hank’s balanced salt solution, then incubated with Alexa Fluor 488 Goat anti Mouse IgG secondary antibodies for 1 hour at RT. Samples were mounted with Prolong Gold anti-fade reagent with 4’6-diamidino-2-phenylindole (DAPI) and imaged with a fluorescence microscope (Olympus IX71).

### Statistical analysis

Statistical analysis was performed using Prism 6.0 (GraphPad Inc, San Diego). One way ANOVA was used in statistics involving platelet counts, the proportion of RPs, and adherent thromboblasts in zebrafish. Means were compared via the Kruskal-Wallis test as appropriate. Two way ANOVA was used to analyze radiotherapy experiments in mice, H&E/IHC-P of bone marrow and lungs, rhTyrRS (Y341A) at different doses and the effects of different drugs on zebrafish megakaryocyte counts. A p value < 0.05 was considered statistically significant.

## Results

### The platelet counts and proportion of RPs was increased by rhTyrRS (Y341A)

**Figure 1:**
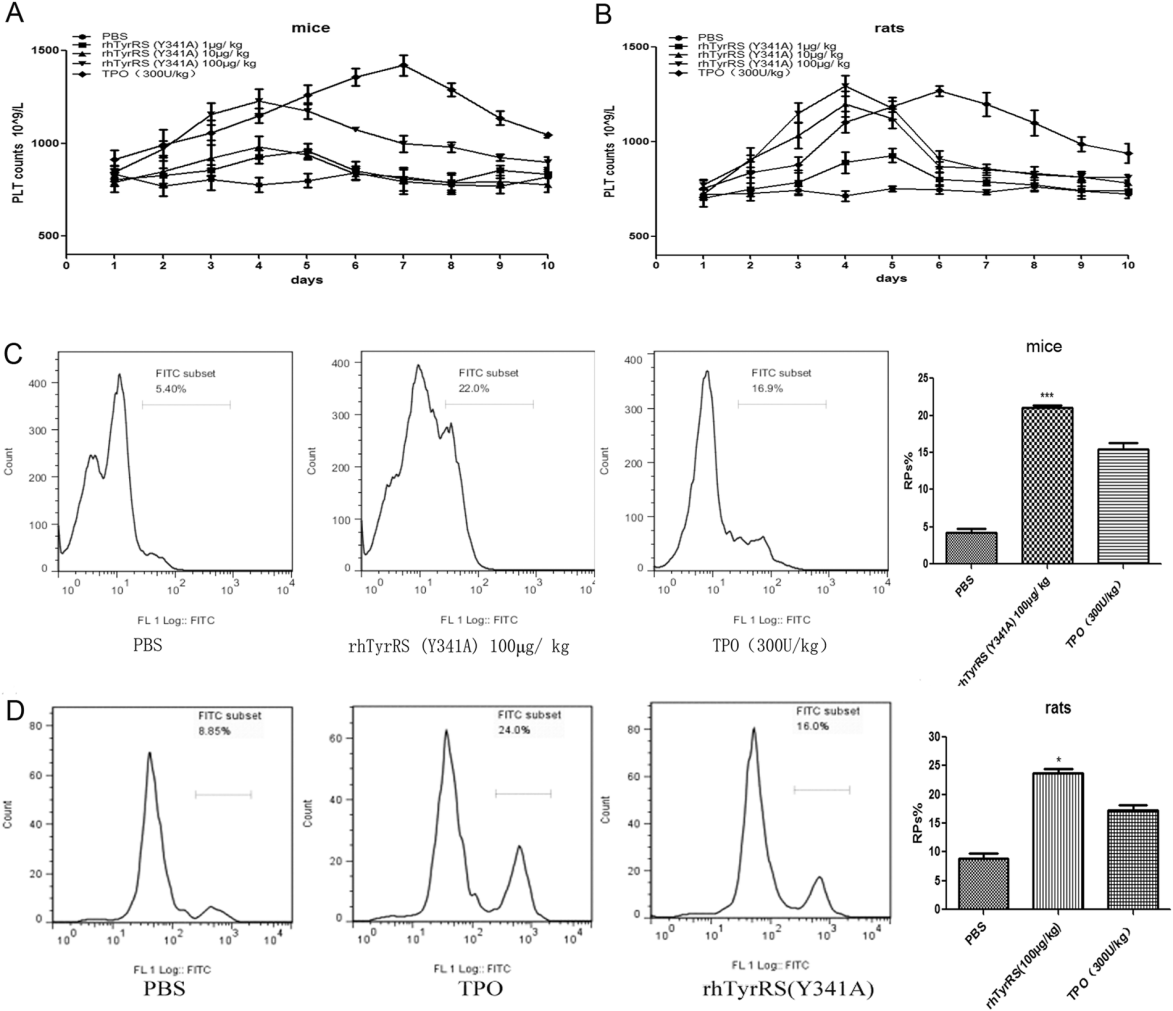
Platelet numbers and the proportion of RPs was increased by rhTyrRS (Y341A). (**A**) Changes in platelet numbers in mice after administration. (**B**) Changes in platelet numbers in rats after administration. High dose rhTyrRS (Y341A) (100 μg/kg) increased the number of platelets faster than TPO in mice and rats. On the first day after administration (fourth day), induced platelet counts reached their the peak. Compared with PBS group (platelet counts 777.0 ± 39.1*10^9^/L), the platelet counts in the high dose rhTyrRS (Y341A) (100 μg/kg) treated group (1227 ± 65.8*10^9^/L) were significantly increased (p < 0.001). In the middle dose rhTyrRS (Y341A) (10 μg/kg) (980 ± 57.4*10^9^/L) group, platelet counts also increased (p < 0.05). From the first to third day of administration, in rhTyrRS (Y341A) treated groups, platelet counts increased dose-dependently (**A**,**B**). After administration of rhTyrRS (Y341A) (100 μg/kg), TPO (300 U/kg), or PBS for 3 consecutive days (n = 6 per group). TO labeled platelets in whole blood were measured by flow cytometry analysis to study percentage of RPs. (**C**) The RP % in mice in the rhTyrRS (Y341A) (100 μg/kg) group (21.0 ± 0.7) was significantly higher than in the PBS group (4.2 ± 1.3) and TPO group (15.4 ± 2.0) (***p < 0.001). There were no significant differences in PBS group and TPO group (ns: p > 0.05) mean ± SEM. (**D**) The same effect was also observed in rats (*p < 0.05).

High dose rhTyrRS (Y341A) (100 μg/kg) increased the number of platelets faster than TPO in mice and rats. On the first day after administration (study day 4), high dose rhTyrRS (Y341A) (100 μg/kg) and middle dose rhTyrRS (Y341A) (10 μg/kg) induced platelet counts to reach their peak. From the first to the third day after administration, platelet counts increased dose-dependently in the rhTyrRS (Y341A) treated groups (Fig. 1A,B).

Flow cytometry analysis showed that mice and rat RPs did stain with TO in every group. Statistical analysis showed that the proportion of RPs in the total platelet count was significantly increased in the rhTyrRS (Y341A) groups compared with the PBS group (Fig. 1C,D) (**P < 0.01, ***P < 0.001). Our results indicated rhTyrRS (Y341A) or TPO accelerated thrombocytopoiesis. Moreover, these results showed that the proportion of RPs in the rhTyrRS (Y341A) (100 μg/kg) treated group were higher than in the TPO treated group in mice and rats, which indicated that rhTyrRS (Y341A) promoted increases in platelet counts more rapidly than TPO.

### RhTyrRS (Y341A) is an effective therapy for mice undergoing radiotherapy-induced thrombocytopenia

**Figure 2:**
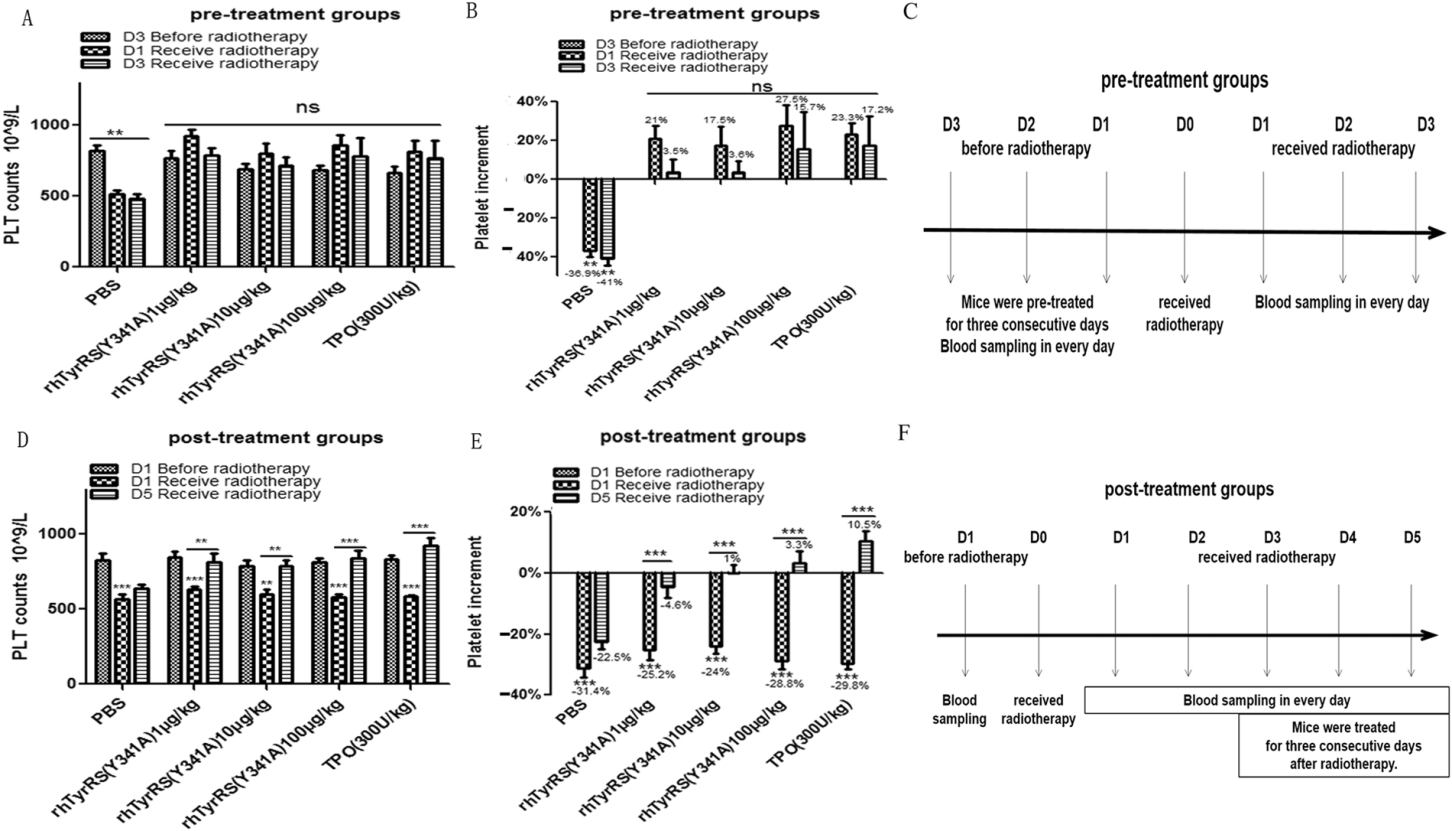
The effect of rhTyrRS (Y341A) on the platelets of mice before and after irradiation. (**A**) Pre-treatment groups. Mice were treated with rhTyrRS (Y341A) for three consecutive days before irradiation. Every series in each group shows the platelet numbers on the third day before radiation, the first day of radiotherapy, and the third day of radiotherapy. (**B**) Incremental changes in platelet numbers in the pre-treatment group. Series at the third day before the radiation was 0%. In the pre-treatment groups, after radiotherapy, the platelet numbers in PBS group were decreased significantly (**P < 0.01), but there were no significant changes in platelet numbers in rhTyrRS (Y341A) or TPO treated groups. (**C**) Shows the administration and blood sampling time point in pre-treatment groups. (**D**) Post-treatment groups, after the third day of irradiation and treatment with rhTyrRS (Y341A) for three consecutive days. Every series in each group shows the platelet numbers on the first day before radiation, the first day of radiotherapy, and the fifth day of radiotherapy (the third day after treatment with rhTyrRS (Y341A)). (**E**) Incremental changes in platelet numbers in the post-treatment group. Series at the first day before the radiation was 0%. In the post-treatment groups, after radiotherapy, the platelet counts were significantly decreased in all groups (**P < 0.01, ***P < 0.001). Compared with the first day of radiotherapy, after treatment, the platelet numbers were significantly increased on the fifth day after radiotherapy in rhTyrRS (Y341A) and TPO groups (**P < 0.01, ***P < 0.001). (**F**) Shows the administration and blood sampling time point in post-treatment groups.

In the pre-treatment groups (Fig. 2A,B) after radiotherapy, platelet numbers in the PBS group were decreased significantly (P < 0.01), but there were no significant changes in platelets numbers in the rhTyrRS (Y341A) treated or TPO treated groups.

In the post-treatment groups (Fig. 2D,E) after radiotherapy, the platelet counts were significantly decreased in all groups (**P < 0.01, ***P < 0.001). Three days after irradiation, mice were treated with rhTyrRS (Y341A) or TPO for three consecutive days. After treatment, the number of platelets in the rhTyrRS (Y341A) and TPO treated groups increased. Compared with the first day after radiotherapy, the platelet numbers were significantly higher on the fifth day in the rhTyrRS (Y341A) and TPO groups (**P < 0.01, ***P < 0.001).

Compared with post-treatment groups, the results showed rhTyrRS (Y341A) pre-treatment might be a better therapeutic regimen because in pre-treatment groups, mice didn’t undergo the thrombocytopenia.

### RhTyrRS (Y341A) did not promote increases in the number of bone marrow megakaryocytes but increased the number of megakaryocytes in the lungs

**Figure 3:**
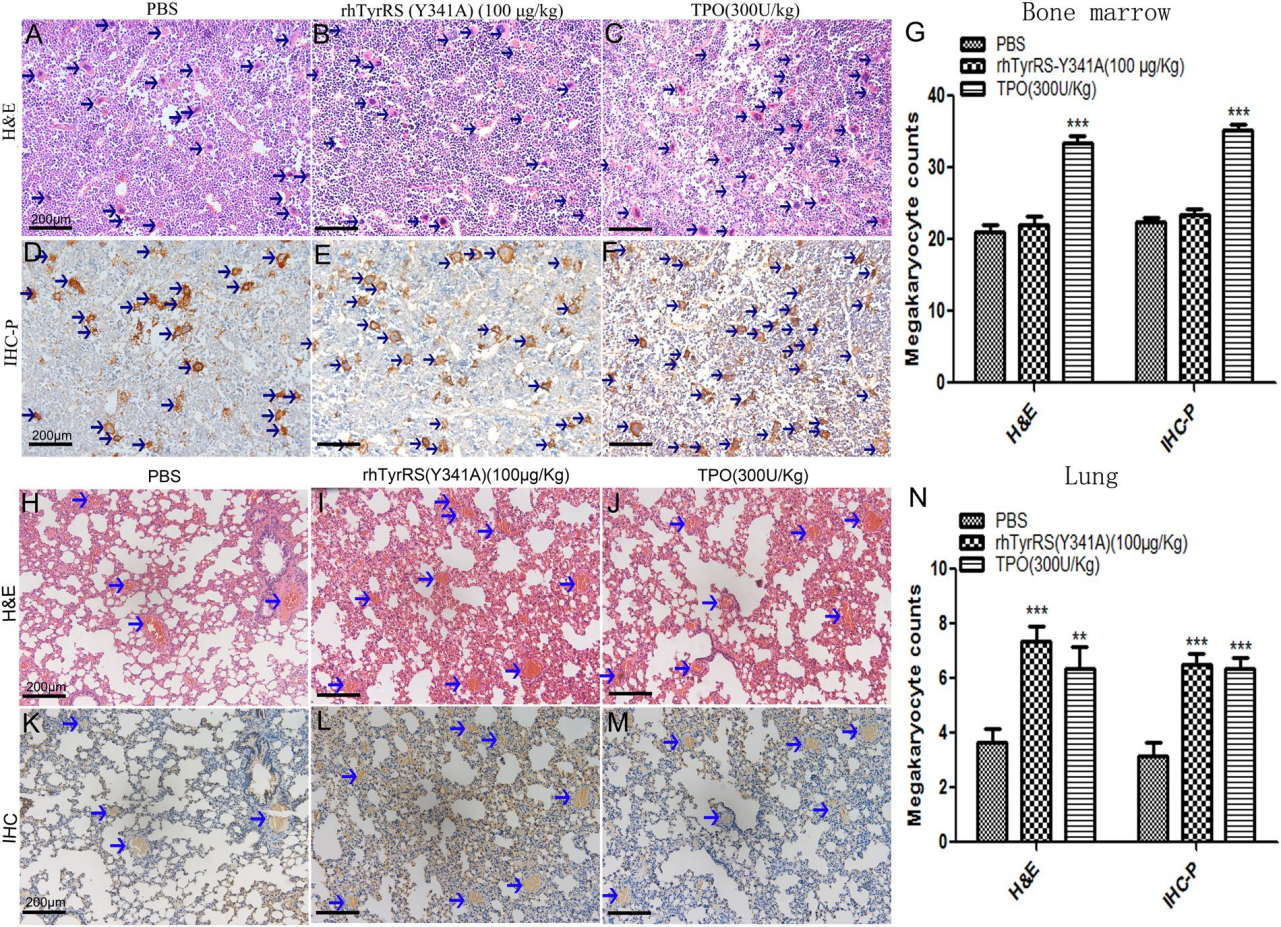
The effects of rhTyrRS(Y341A) on megakaryocytes were studied by immunohistochemistry and (**H**&**E**) staining in the bone marrow and lung. (**H**&**E**) staining showed there was no increase in the number of bone marrow megakaryocyte cells in the (**B**) rhTyrRS (Y341A) (100 μg/kg) group (22.0 ± 1.2) or (**A**) PBS group (21 ± 1.25). However, the number of bone marrow megakaryocyte cells increased significantly in (**C**) the TPO group (33.5 ± 1.0) (***p < 0.001). Immunohistochemistry of bone marrow megakaryocytes (CD41^+^) also showed there was no increase in the number of bone marrow megakaryocyte cells in the (**E**) rhTyrRS (Y341A) (100 μg/kg) (23.3 ± 0.8) or (**D**) PBS group (22.3 ± 0 0.67), while the number of bone marrow megakaryocyte cells increased significantly in the (**F**) TPO group (35.2 ± 0.9) (***p < 0.001), mean ± SEM. (**G**) Statistics on the number of bone marrow megakaryocytes in H&E staining and immunohistochemistry (***P < 0.001). Lung (**H**&**E**) staining showed there was no increase in the number of lung megakaryocyte cells in the (**H**) PBS group (3.67 ± 0.49), while the number of lung megakaryocyte cells increased significantly in the (**I**) rhTyrRS (Y341A) (100 μg/kg) (7.33 ± 0.56) and (**J**) TPO group (6.33 ± 0.84) (***p < 0.001,**p < 0.01). Immunohistochemistry of lung megakaryocytes (CD41^+^) also showed there was no increase in the number of lung megakaryocyte cells in the (**K**) PBS group (3.17 ± 0.48), while the number of lung megakaryocyte cells increased significantly in the (**L**) rhTyrRS (Y341A) (100 μg/kg) (6.5 ± 0.43) and (**M**) TPO group (6.3 ± 0.42) (***p < 0.001), mean ± SEM. (**N**) Statistics on the megakaryocyte numbers in lungs using H&E staining and immunohistochemistry (***P < 0.001, **p < 0.01).

Pathologic analysis showed that there were no increases in the number of bone marrow megakaryocytes in the PBS (Fig. 3A,D) and rhTyrRS (Y341A) treated groups (Fig. 3B,E), while the number of bone marrow megakaryocytes was increased in TPO group (Fig. 3C,F). However, megakaryocyte counts in the lungs of mice in the rhTyrRS (Y341A) (Fig. 3I,L) and TPO treated groups (Fig. 3J,M) were higher than the PBS treated group (Fig. 3H,K). These results demonstrate that rhTyrRS (Y341A) can increase the number of platelets in the peripheral blood without increasing the number of megakaryocytes in the bone marrow. The mechanism by which rhTyrRS (Y341A) increases platelet counts is different than the mechanism by which TPO acts.

Statistical results (Fig. 3N) showed that the number of megakaryocytes in the lungs of mice in the rhTyrRS (Y341A) (100 g/kg) and TPO (300 U/kg) group was significantly higher than in the PBS group. These results show that the lungs are a primary site of terminal platelet production^1^.

### RhTyrRS (Y341A) impacts zebrafish thrombocytes

**Figure 4:**
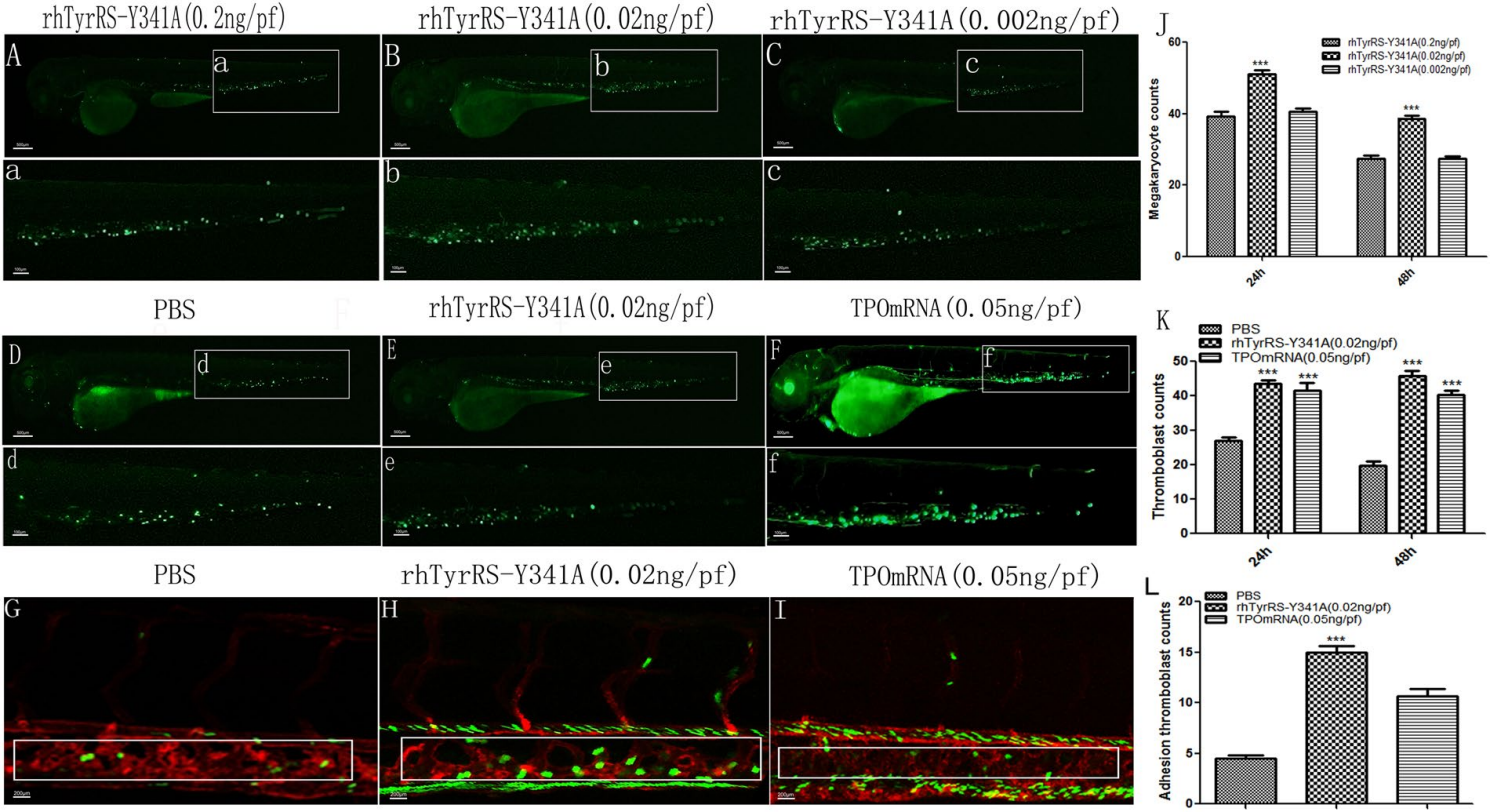
The effect of rhTyrRS (Y341A) on zebrafish thromboblasts. Green areas represent the thromboblasts labeled by GFP in zebrafish. The changes in thromboblast numbers in zebrafish at 24 h after micro-injection of rhTyrRS (Y341A) (0.002, 0.02, or 0.2 ng per fish) are shown in (**A**,**B**,**C**). (**J**) Statistics on the number of thromboblasts in zebrafish at different time points (P < 0.001); the abscissa indicates the observation time after microinjection. The ordinate indicates the number of thromboblasts. The changes in thromboblast counts in the PBS, rhTyrRS (Y341A) (2 × 10^−2^ ng per fish) group, and TPO mRNA (0.05 ng per fish) group at 24 h after micro-injection are shown in (**D**,**E**,**F**). (**K**) Statistics on the number of thromboblasts in zebrafish at different time points (P < 0.001). The abscissa indicates the observation time after microinjection. The ordinate indicates the number of thromboblasts. Laser confocal microscope observation of thromboblast adhesion to vascular endothelial cells 24 h after micro-injection. (**G**) PBS group. (**H**) rhTyrRS (Y341A) (0.02 ng per fish) group. (**I**) TPO mRNA (0.05 ng per fish) group. (**L**) Statistics on the number of thromboblasts adhering to vascular endothelial cells in caudal hematopoietic tissue. The abscissa indicates the observation time after micro-injection; the ordinate indicates the number of thromboblast adhesion (***P < 0.001).

In zebrafish, the thromboblast functions similar to the human megakaryoctye. Zebrafish were administered different doses of rhTyrRS (Y341A) via microinjection. We found that different doses of rhTyrRS (Y341A) differentially affect the number of thromboblasts in zebrafish. The number of thromboblasts at 24 and 48 h was highest with a dose of 0.02 ng per fish of rhTyrRS (Y341A) (Fig. 4J).

Compared with the PBS group, the number of thromboblasts in zebrafish were significantly increased in rhTyrRS (Y341A) and TPO mRNA groups (***P < 0.001). The number of thromboblasts at different time points did not change with the microinjection of PBS (Fig. 4K).

RhTyrRS (Y341A) promoted thromboblast adhesion to vascular endothelial cells. The adhesion of thromboblasts to vascular endothelial cells in zebrafish caudal hematopoietic tissue (CHT) after treatment with PBS, rhTyrRS (Y341A) (0.02 ng per fish), or TPO mRNA (0.05 ng per fish) was further investigated via confocal microscopy.

We found that the number of thromboblasts adhering to the zebrafish caudal hematopoietic tissue (CHT) in the rhTyrRS (Y341A) group (Fig. 4H) was significantly higher than in the PBS (negative control) (Fig. 4G) and TPO mRNA group (positive control) (Fig. 4I).

### RhTyrRS (Y341A) promotes NF-κB pathway activation in HUVECs

**Figure 5:**
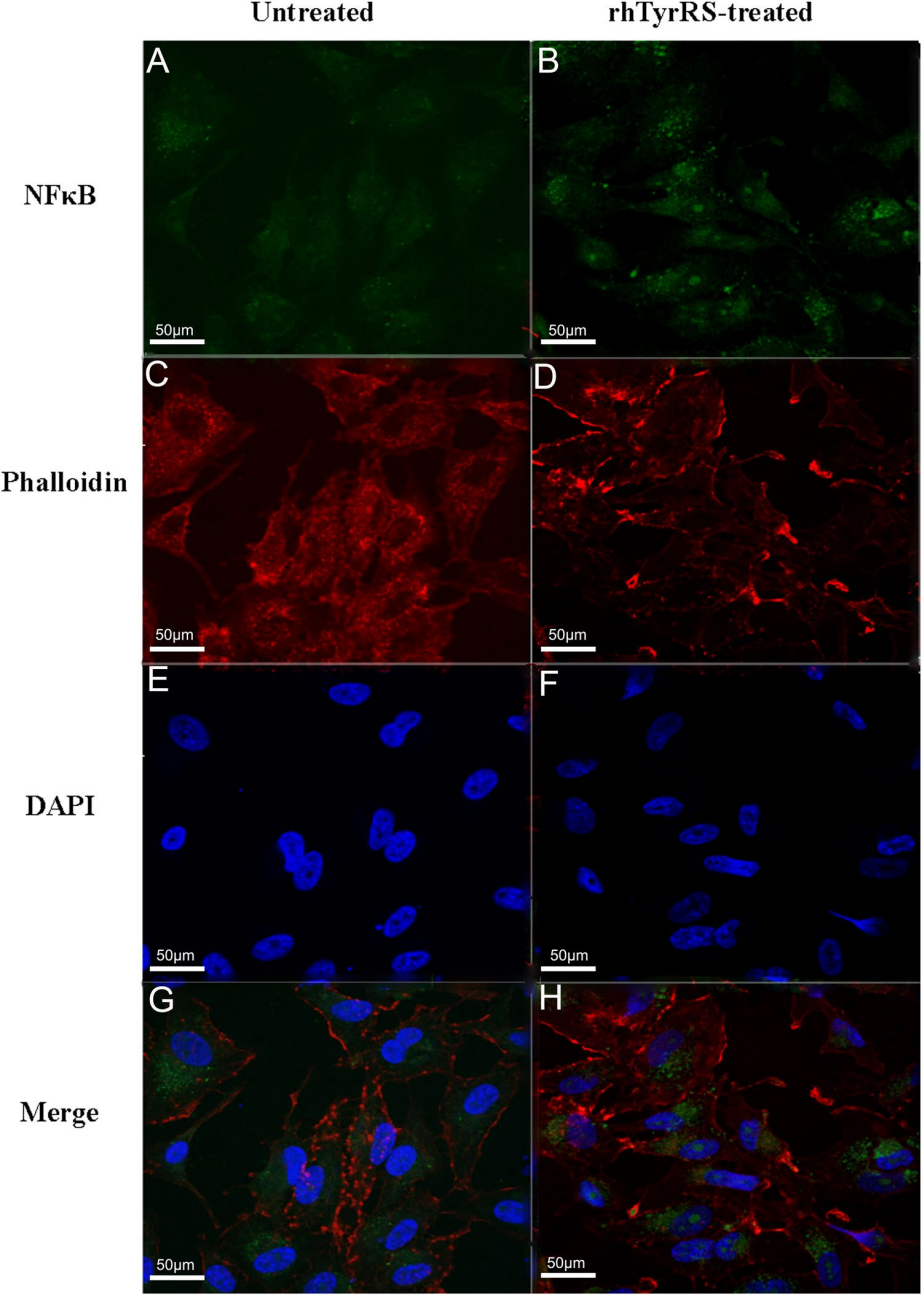
rhTyrRS (Y341A) promotes NF-κ B pathway activation. 10 nM rhTyrRS (Y341A) or PBS was added to cultured HUVECs, which were stimulated for two hours. The signal for NF-κB in the nucleus (indicative of pathway activation) in the rhTyrRS (Y341A) group was increased, while NF-κB largely remained distributed within the cytoplasm in the PBS group.

NF-κB was labeled with a green fluorescent protein (Fig. 5A,B). Cytoplasmic F-actin was stained by phalloidin (red fluorescence) (Fig. 5C,D). The nucleus was stained with DAPI (blue fluorescence) (Fig. 5E,F). After 10 nM rhTyrRS (Y341A) was added to the cultured HUVECs and incubated for 2 h, signal for NF-κB in the nucleus (indicative of pathway activation) increased in the rhTyrRS (Y341A) group, while NF-κB largely remained distributed within the cell cytoplasm in the PBS group (Fig. 5G,H).

## Discussion

In our research, we found rhTyrRS (Y341A) induced thrombocytopoiesis more rapidly than TPO. However, the mechanisms by which these acted were very different.

In Fig. 1, after three days of treatment, the platelet numbers in the rhTyrRS (Y341A) treated group (100 μg/kg) were higher than in the TPO treated group, which showed that rhTyrRS (Y341A) acted faster than TPO. We speculated that might be due to the different mechanisms by which rhTyrRS (Y341A) and TPO act. The rhTyrRS (Y341A) helps mobilize differentiated megakaryocytes to the peripheral blood and accelerates adhesion to vascular endothelial cells to induce thrombocytopoiesis. TPO, on the other hand, accelerates adult stem cell differentiation to megakaryocytes in the bone marrow, resulting in an increase in the number megakaryocytes to induce thrombocytopoiesis. TPO is important in the self-renewal of multi-potent progenitors, and studies indicate that TPO stimulates 5 to 6 megakaryocyte endreduplication^25^, while adult stem cell differentiation to megakaryocytes requires more time. Therefore, the number of platelets in the rhTyrRS (Y341A) (100 µg/kg) group increased rapidly, while numbers in the TPO group increased more slowly. In this study, we found that rhTyrRS (Y341A) promotes platelet number increases more rapidly than TPO, but there was no increase in the number of megakaryocytes within the bone marrow (Fig. 3).

Our experimental results showed that after drug administration, rhTyrRS (Y341A) group megakaryocytes in the bone marrow were not significantly increased, but the number of pulmonary megakaryocytes increased significantly compared to the PBS group. These findings indicate that rhTyrRS (Y341A) can mobilize megakaryocytes, directing them into the lungs, which also shows that the pulmonary system plays an important role in thrombocytopoiesis^1^.

We first discovered that rhTyrRS (Y341A) has effects on thromboblasts in zebrafish. We also found that the number of GFP marked thromboblasts in the peripheral blood began to wane at 72 h, because the drug had been metabolized and its action started to weaken. At the same time, thromboblasts in the peripheral blood rapidly release new platelets, recognized as reticulated platelets, thus increasing the platelet count. The migration of thromboblasts is an important step in thrombocytopoiesis^36,37^. Laser confocal experiments were used to explain the effect of rhTyrRS (Y341A) on thromboblast mobility. We found that rhTyrRS (Y341A) possesses the ability to promote thromboblast migration and adhere to the caudal venous plexus of endothelial cells. Part of the thromboblast will migrate within the caudal hematopoietic tissue. After treatment, the number of adherent thromboblasts was increased, and the number of thromboblasts in the peripheral blood was also increased. However, the number of adherent thromboblast in the PBS and TPO mRNA groups were significantly lower than in the rhTyrRS (Y341A) group. Combined with immunohistochemistry and histological analysis, we can speculate that rhTyrRS (Y341A) plays a role in promoting the adhesion of megakaryocytes or thromboblasts to venous endothelial cells. Megakaryocyte and thromboblasts underwent accelerated differentiation with rhTyrRS treatment to generate more platelets.

After rhTyrRS (Y341A) administration, peripheral blood platelet numbers were increased in all the animal experiments, which was also demonstrated by flow cytometry. After treatment, the numbers of RPs in the rhTyrRS (Y341A) (100 µg/kg) group were higher than in the TPO and PBS groups. This demonstrated that RPs numbers were increased by rhTyrRS (Y341A), eventually resulting in an increase in the number of mature megakaryocytes.

MiniTyrRS in human beings would specifically bind to IL-8 receptor A on polymorphonuclear leukocytes (PMNs) to induce PMN migration; these effects are concentration dependent and very similar to IL-8 in the CXC chemokine subfamily^12,38^. In contrast, in experiments applying different rhTyrRS (Y341A) doses in zebrafish, we found that different doses produce unique effects. Excessively high concentrations of rhTyrRS (Y341A) ultimately inhibit the production of platelets.

Studies have shown that Tumor necrosis factor α (TNF-α) promotes ERK 1/2, p38, and JNK 1/2 phosphorylation and NF-κB nuclear entry to induce VCAM-1 expression and ultimately promote cell adhesion^39,40^. IL-8 may also promote cell adhesion through the NF-κB signaling pathway. The function and cytokine-like structure of rhTyrRS (Y341A) is very similar to IL-8. Therefore, we speculated that VCAM-1 expression is promoted by rhTyrRS (Y341A) through the NF-κB signaling pathway. Accordingly, megakaryocytes are prompted to migrate and adhere to vascular endothelial cells, such that the number of megakaryocytes would increase. Previous reports suggest that pre-treatment with TPO in patients receiving high-dose chemotherapy may result in less severe thrombocytopenia. In the current study, we provide compelling evidence that rhTyrRS (Y341A) is an effective therapy for mice undergoing radiotherapy-induced thrombocytopenia.

In summary, our research showed that rhTyrRS (Y341A) effectively increased the number of platelets in peripheral blood, but the number of megakaryocytes in bone marrow was not increased. Immunohistochemistry and H&E staining showed that the number of pulmonary mature megakaryocytes was significantly increased in rhTyrRS (Y341A) treated group; confocal microscopy results showed rhTyrRS (Y341A) promoted the migration and adhesion of megakaryocytes, which suggested that rhTyrRS (Y341A) promotes megakaryocytes in bone marrow and peripheral blood migrate to lung endothelial cells. The rhTyrRS (Y341A) may be an effective medicine which could be used to treat patients suffering from life-threatening thrombocytopenia or bone marrow failure.
